# 3,3′-Diindolylmethane Promotes BDNF and Antioxidant Enzyme Formation via TrkB/Akt Pathway Activation for Neuroprotection against Oxidative Stress-Induced Apoptosis in Hippocampal Neuronal Cells

**DOI:** 10.3390/antiox9010003

**Published:** 2019-12-18

**Authors:** Bo Dam Lee, Jae-Myung Yoo, Seong Yeon Baek, Fu Yi Li, Dai-Eun Sok, Mee Ree Kim

**Affiliations:** 1 Department of Food and Nutrition, Chungnam National University, Daejeon 34134, Korea; bodam_lee@naver.com (B.D.L.); qor7683@naver.com (S.Y.B.); kaihuadouer@naver.com (F.Y.L.); 2 Korean Medicine-Application Center, Korea Institute of Oriental Medicine, Daegu 41062, Korea; jmyoo@cnu.ac.kr; 3 Korean Medicine R&D Team 1, National Institute for Korean Medicine Development, Gyeongsan 38540, Korea; 4 College of Pharmacy, Chungnam National University, Daejeon 34134, Korea; daesok@cnu.ac.kr

**Keywords:** 3,3′-diindolylmethane, antioxidant enzymes, brain-derived neurotrophic factor, hippocampal neuronal cells, neurodegenerative disease

## Abstract

3,3′-Diindolylmethane (DIM), a metabolite of indole-3-carbinol present in Brassicaceae vegetables, possesses various health-promoting effects. Nonetheless, the effect of DIM on neurodegenerative diseases has not been elucidated clearly. In this study, we hypothesized DIM may protect neuronal cells against oxidative stress-induced apoptosis by promoting the formation of brain-derived neurotrophic factor (BDNF) and antioxidant enzymes through stabilizing the activation of the tropomyosin-related kinase receptor B (TrkB) cascade and we investigated the effect of DIM on oxidative stress-mediated neurodegenerative models. DIM protected neuronal cells against oxidative stress-induced apoptosis by regulating the expression of apoptosis-related proteins in glutamate-treated HT-22 cells. Additionally, DIM improved the expression of BDNF and antioxidant enzymes, such as heme oxygenase-1, glutamate-cysteine ligase catalytic subunit, and NAD(P)H quinine oxidoreductase-1, by promoting the activation of the TrkB/protein kinase B (Akt) pathway in the cells. Consistent with in vitro studies, DIM attenuated memory impairment by protecting hippocampal neuronal cells against oxidative damage in scopolamine-treated mice. Conclusionally, DIM exerted neuroprotective and antioxidant actions through the activation of both BDNF production and antioxidant enzyme formation in accordance with the TrkB/Akt pathway in neuronal cells. Such an effect of DIM may provide information for the application of DIM in the prevention of and therapy for neurodegenerative diseases.

## 1. Introduction

3,3′-Diindolylmethane (DIM) is a metabolite of indole-3-carbinol, which is present in Brassicaceae vegetables containing glucosinolates (206–3895 mg/kg) [1]. Hydrolysis products of glucosinolates, such as isothiocyanates and indol-3-carbinol, are well known for exerting health-promoting effects, including for neurodegenerative diseases [2]. DIM, a dimer of indole-3-carbinol that is converted in the acidic conditions of stomach after intake [3], is known to possess beneficial effects especially in antioxidant [4], anti-cancer [5], and hepatoprotective actions [6]. Recently, it was reported that DIM could protect neuronal cells against inflammation [7,8] and ischemia [7,8] in brain tissue. Moreover, DIM at the doses used did not show any serious side effects in healthy volunteers [9]. Nonetheless, the effect of DIM on neurodegenerative diseases has not been elucidated clearly.

Neurodegenerative diseases, which are increasing along with the extension of life spans and the occurrence of metabolic diseases, are known to be associated with the generation of reactive oxygen species (ROS) [10], which are responsible for the apoptosis of neuronal cells [11,12]. Therefore, the regulation of ROS generation is suggested as a key target for the treatment or prevention of neurodegenerative diseases. Brain-derived neurotrophic factor (BDNF), which is mainly produced from the central nervous system [13,14,15] and is concerned with the preservation of neuronal cells in in vivo systems, is known to play a crucial role [16]; it participates in the proliferation and differentiation of neuronal cells through the activation of tropomyosin-related kinase receptor B (TrkB) [17]. In addition, BDNF protects neuronal cells against oxidative stress [16,18]. Recently, it was observed that some specific antioxidant compounds ameliorate scopolamine-induced memory impairment by promoting BDNF production and suppressing oxidative damage in scopolamine-exposed mice [11,12,19]. Therefore, the regulation of BDNF formation may be a key point for the treatment or prevention of neurodegenerative diseases such as Alzheimer’s disease.

Currently, inhibitors of acetylcholinesterase (AChE) have been used for treatment of Alzheimer’s disease [20]. However, these inhibitors only delay the progression of Alzheimer’s disease and also produce side effects [20]. In this respect, phytochemicals derived from vegetables may be preferable alternatives for the prevention or treatment of neurodegenerative diseases such as Alzheimer’s disease.

In this study, we investigated whether DIM was able to protect hippocampal neuronal cells against oxidative stress-induced apoptosis, and then we examined how DIM protects neuronal cells against oxidative stress-induced apoptosis in both in vitro and in vivo models. Herein, we report that the neuroprotective and antioxidant properties of DIM are closely associated with the production of BDNF and antioxidant enzymes by activation of the pathways involving TrkB/protein kinase B (Akt) in hippocampal neuronal cells. Such an effect of DIM may help to address the neuroprotective action of indole-3-carbinol and some vegetables of the brassicaceae family and may provide information for the application of DIM as a therapeutic or preventive supplement for neurodegenerative diseases.

## 2. Materials and Methods

### 2.1. Materials

Antibiotics, FBS, 0.25% trypsin-EDTA, and 2,7-dichlorofluorescein diacetate (DCFDA) were procured from Invitrogen (Carlsbad, CA, USA). Dulbecco’s modified eagle medium (DMEM), 1× PBS, and 1× Tris buffered saline (TBS) were purchased from Welgene, Inc. (Gyeongsan, Gyeongbuk, Korea). EZ-Cytox cell viability assay kit was obtained from DAEIL Lab (Seoul, Korea). DIM, K252a (a TrkB inhibitor), and MK-2206 (a specific Akt inhibitor) were procured from Cayman Chemical Company (Ann Arbor, MI, USA). Specific antibodies against cytochrome c (ab90529), glutamate-cysteine ligase catalytic subunit (GCLC, ab53179), and NAD(P)H quinone oxidoreductase-1 (NQO-1, ab34173) were purchased from Abcam, Inc. (Cambridge, UK). A specific antibody against BDNF (sc-65514) was obtained from Santa Cruz Biotechnology, Inc. (Dallas, TX, USA). Specific antibodies against apoptosis-inducing factor (AIF, #4642), B-cell lymphoma 2 (Bcl-2, #3498), Bcl-2-associated X protein (Bax, #2772), cleaved caspase-3 (#9661), heme oxygenase-1 (HO-1, #70081), NF-E2-related factor-2 (Nrf2, #12721), phospho-Akt (#9271), phospho-cAMP response element-binding protein (CREB, #9198), phospho-TrkB (#4619), and β-actin (#4970) as well as a horseradish peroxidase-conjugated IgG secondary antibody were procured from Cell Signaling Technology (Beverly, MA, USA). Muse Annexin V and Dead Cell Assay Kits were purchased from Merck Millipore, Inc. (Darmstadt, Germany). All other chemicals which were used in this study were analytical grade.

### 2.2. Animals

ICR mice [21] were obtained from Nara Biotech Co. (Pyeongteak, Korea). Their gender, age, and body weight were male, 6 weeks, and 25–30 g. The mice were housed in cages (five mice/cage) under specific pathogen-free conditions (21–24 °C and 40–60% relative humidity) with a 12 h light/dark cycle. In addition, they were given free access to standard rodent food (Orientbio Inc., Seongnam, Korea) and water. All animal experiments were approved by the Committee of Animal Care and Experiment of Chungnam National University (Daejeon, Korea) with a reference number (CNU-00973) and carried out following the guidelines of the Animal Care and Use Committee at Chungnam National University.

### 2.3. Behavior Tests

#### 2.3.1. Morris Water Maze Test

The cognitive ability of mice was evaluated by Morris water maze test, which was reported previously [12]. One hour before the Morris water maze test, mice were administered with DIM (0, 10, or 20 mg/kg, orally). After three consecutive days, the mice were administered with scopolamine (2 mg/kg, i.p.) after the DIM treatment.

#### 2.3.2. Passive Avoidance Test

The cognitive ability of the mice was evaluated by the passive avoidance test, which was reported previously [12]. One hour before the acquisition trial, the mice were orally administered with DIM before the scopolamine challenge.

### 2.4. Analysis of Antioxidant or Neurobiological Biomarkers in Brain Tissues

#### 2.4.1. Determination of Lipid Peroxidation

Lipid peroxidation was determined following a method reported previously [22]. Briefly, brains isolated from sacrificed mice were homogenized in ice-cold 1× PBS, and then the homogenates were centrifuged (17,000× *g* at 4 °C) for 10 min. The supernatant (100 μL) was mixed with 50 μL of 20% aqueous trichloroacetic acid and 100 μL of 0.67% aqueous thiobarbituric acid, boiled for 10 min, and then centrifuged (60× *g*, room temperature (RT) for 30 min. The absorbance of the supernatant was measured at 532 nm using a microplate reader (DU650, Beckman Coulter, Brea, CA, USA).

#### 2.4.2. Measurement of Glutathione

Glutathione (GSH) level in brain tissues or cell pellets was analyzed using a Quanticrom Glutathione Assay Kit obtained from BioAssay Systems (Hayward, CA, USA) according to the manufacturer’s protocol.

#### 2.4.3. Activities of Glutathione Reductase and Glutathione Peroxidase

The activities of glutathione reductase (GR) and glutathione peroxidase (GPx) in brain tissues were determined as described previously [19].

#### 2.4.4. Activities of Acetylcholinesterase and Choline Acetyltransferase

The activities of acetylcholinesterase (AChE) and choline acetyltransferase (ChAT) in brain tissues were determined as described previously [19].

### 2.5. Hematoxylin and Eosin Staining Assay

Histological analysis was conducted by following a modification of a method reported previously [23]. Briefly, deparaffinized brain tissue slices were stained with hematoxylin-eosin. The stained tissue slices were embedded with mounting solution. Histological changes of the brain tissues were observed under a light microscope with 200× magnification.

### 2.6. Cell Culture

The HT-22 cell line was procured from Medifron (Seoul, Korea), and HT-22 cells were maintained in DMEM medium with 10% FBS, 100 units/mL of penicillin, and 100 μg/mL of streptomycin at 37 °C in a humidified atmosphere of 5% CO_2_. Passages three to 11 of the cells were used in this study. All the in vitro studies included a vehicle control group (0.1% dimethyl sulfoxide).

### 2.7. Cell Viability Assay

Cell viability was assessed according to a previous method [24]. HT-22 cells were preincubated with or without DIM (0–80 μM) for 30 min before glutamate challenge. After 12 h, cell viability was determined using the EZ-Cytox cell viability assay kit following the manufacturer’s instructions.

### 2.8. Measurement of Intracellular ROS Level

The intracellular ROS level was assessed according to a previous method [24]. After glutamate treatment, HT-22 cells were stained with 10 μM DCFDA in Hank’s balanced salt solution for 30 min in the darkness, and the fluorescence (an excitation wavelength of 485 nm and an emission wavelength of 525 nm) was measured by a microplate reader (Beckman Coulter DTX 880 Multimode Detector, Brea, CA, USA).

### 2.9. Extraction of Nuclear and Cytosolic Protein

Cytosolic and nuclear proteins were fractionated using a Nuclear Extraction Kit (Cayman Chemical, Ann Arbor, MI, USA) following the manufacturer instructions.

### 2.10. Immunoblot Analysis

Immunoblot analysis was carried out according to a previous method [12]. Briefly, blotted proteins on PVDF membrane were visualized by a WEST One western blot detection system (iNtRON Biotechnology, Inc., Gyeonggi-do, Korea). All the target protein levels were compared with loading control levels (β-actin or Lamin B), and the results were expressed as a density ratio of each protein, identified by a protein standard size marker (BIOFACT, Daejeon, Korea). The relative density of the protein expression was quantitated by Matrox Inspector software (version 2.1 for Windows; Matrox Electronic Systems Ltd., Dorval, Quebec, Canada).

### 2.11. Statistical Analysis

The experimental results were expressed as the mean ± SD for the in vitro data and as the mean ± SEM for the in vivo data. One-way analysis of variance (ANOVA) was used for multiple comparisons (GraphPad Prism version 5.03 for Windows, San Diego, CA, USA). The Dunnett test and the Tukey’s test were applied for significant variations between treated groups. Differences at the * *p* < 0.05 and ** *p* < 0.01 levels were considered statistically significant.

## 3. Results

### 3.1. Neuroprotective Effect of 3,3′-Diindolylmethane on Glutamate-Treated HT-22 Cells

**Figure 1:**
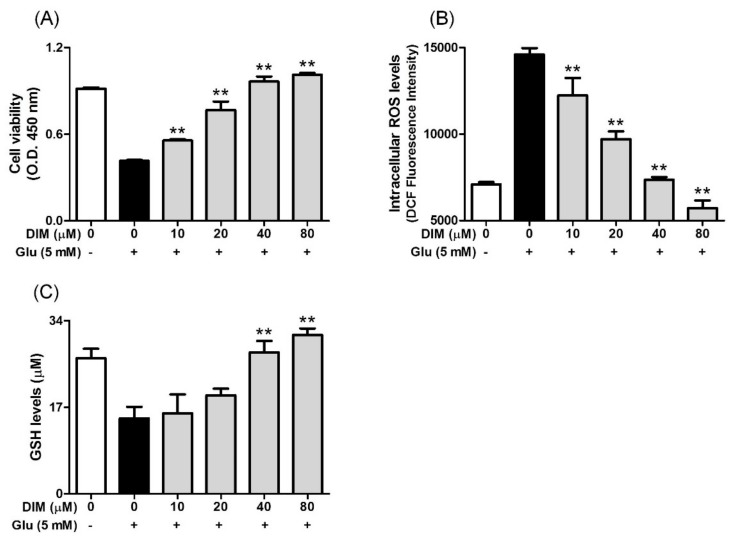
Effect of 3,3′-diindolylmethane (DIM) on glutamate-induced cytotoxicity, and reduction of ROS and glutathione levels in HT-22 cells. HT-22 cells, seeded on a 96 well-plates and incubated for 24 h, were incubated with or without DIM (0–80 μM) for 30 min before glutamate challenge (5 mM). After 12 h, cell viability, ROS level, and GSH level were measured as described in Materials and Methods. (**A**) Cell viability, (**B**) ROS level, and (**C**) GSH level. Data are the mean ± SD values of triple determinations. ** *p* < 0.01 versus glutamate-treated group. – is absence, + is presence.

DIM possesses some beneficial properties, such as antioxidant action [4] and neuroprotective action [7,8]. Considering this, we investigated the effect of DIM on glutamate-induced cytotoxicity in HT-22 cells. When HT-22 cells were incubated with DIM prior to glutamate exposure, DIM at the concentrations used (10–80 μM) enhanced cell viability up to a concentration of 80 μM (Figure 1A). In addition, DIM dose-dependently reduced the ROS level and also restored the GSH level in the cells (Figure 1B,C). A DIM concentration of 40 μM was sufficient to restore the level of ROS or GSH to that of the control group. These results suggest that DIM exerts neuroprotective activity by suppressing oxidative stress in hippocampal neuronal cells.

### 3.2. Inhibitory Effect of 3,3′-Diindolylmethane on Oxidative Stress-Induced Apoptosis

**Figure 2:**
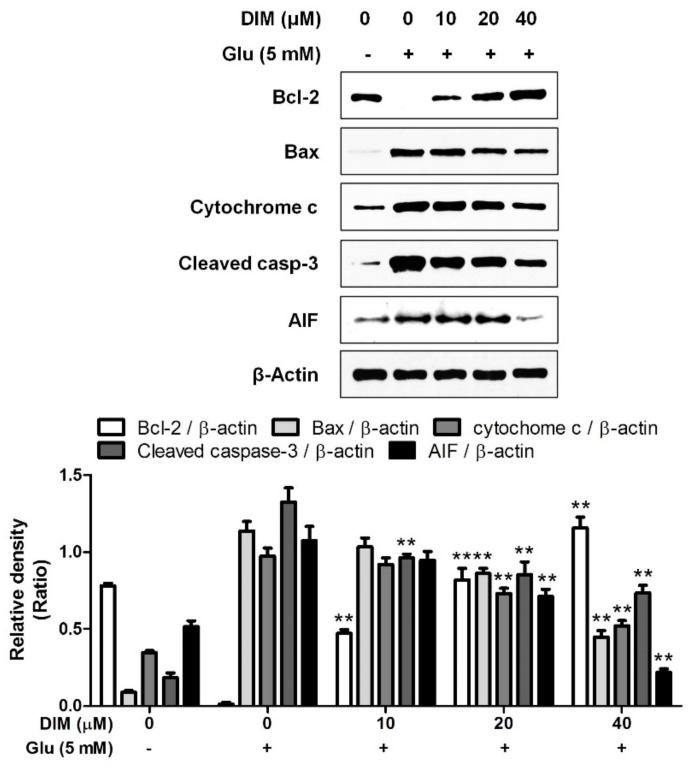
Suppressive effect of 3,3′-diindolylmethane on glutamate-induced apoptosis in HT-22 cells. HT-22 cells were seeded on a 60 mm dish, and then incubated for 24 h. The cells were challenged with glutamate after preincubation with or without DIM (0–40 μM) for 30 min. After 12 h, the expression of Bcl-2, Bax, cytochrome c, cleaved caspase-3, AIF, or β-actin was examined as described in Materials and Methods. The data were contained from three independent experiments. ** *p* < 0.01 versus glutamate-treated group. – is absence, + is presence.

After we found that DIM protects hippocampal neuronal cells against oxidative stress, we investigated the effect of DIM on the expression of apoptosis-related proteins in oxidative stress-exposed neuronal cells. In the previous report, we found that the elevation of ROS formation activated apoptosis signaling pathway in neuronal cells [12]. DIM downregulated the expression of pro-apoptotic factors such as Bax, cytochrome c, cleaved caspase-3, and AIF, whereas it upregulated that of Bcl-2, an anti-apoptotic factor (Figure 2). These findings suggest that DIM protects hippocampal neuronal cells against oxidative stress-induced apoptosis by regulating the expression of apoptosis-related proteins.

### 3.3. Activatory Effect of 3,3′-Diindolylmethane on Both TrkB/CREB/BDNF Pathway and Akt/Nrf2/ARE Pathway

**Figure 3:**
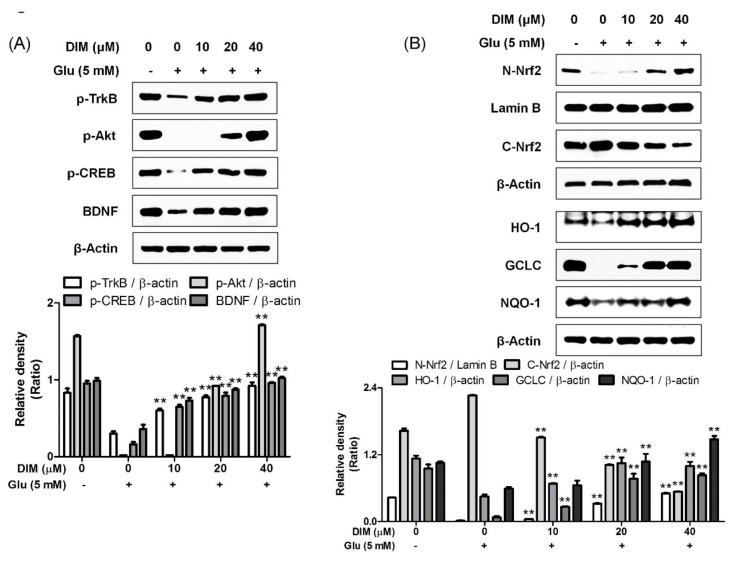
Effect of 3,3′-diindolylmethane on phosphorylation of TrkB, Akt, or CREB, and expression of BDNF, Nrf2, or antioxidant enzymes. The experiments were performed as described in the Figure 2 legend. The data were obtained from three independent experiments. (**A**) p-TrkB, p-Akt, p-CREB, and BDNF and (**B**) nuclear and cytosolic Nrf2, HO-1, GCLC, and NQO-1. ** *p* < 0.01 versus glutamate-treated group. − is absence, + is presence.

It was reported that activation of the TrkB/CREB/BDNF pathway and the Akt/Nrf2/antioxidant response element (ARE) pathway contributed to the neuroprotective action of *N*-acetyl serotonin [12]; therefore, we supposed that both pathways could be activated by DIM, which was found to protect hippocampal neuronal cells against oxidative stress-induced apoptosis. DIM dose-dependently improved the expression of p-TrkB, p-CREB, BDNF, or p-Akt and antioxidant enzymes such as HO-1, GCLC, and NQO-1 in glutamate-treated HT-22 cells (Figure 3). In addition, DIM also promoted the nuclear translocation of Nrf2 in glutamate-treated HT-22 cells. Especially, DIM at 40 µM almost fully restored the expression of all the proteins. Based on these results, we suppose that DIM is able to promote the activation of both the TrkB/CREB/BDNF pathway and the Akt/Nrf2/ARE pathway in oxidative stress-exposed HT-22 cells. Therefore, the neuroprotective action of DIM may be closely associated with the activation of both signaling pathways.

### 3.4. Suppressive Effects of K252a and MK-2206 on Neuroprotective Action of 3,3′-Diindolylmethane

**Figure 4:**
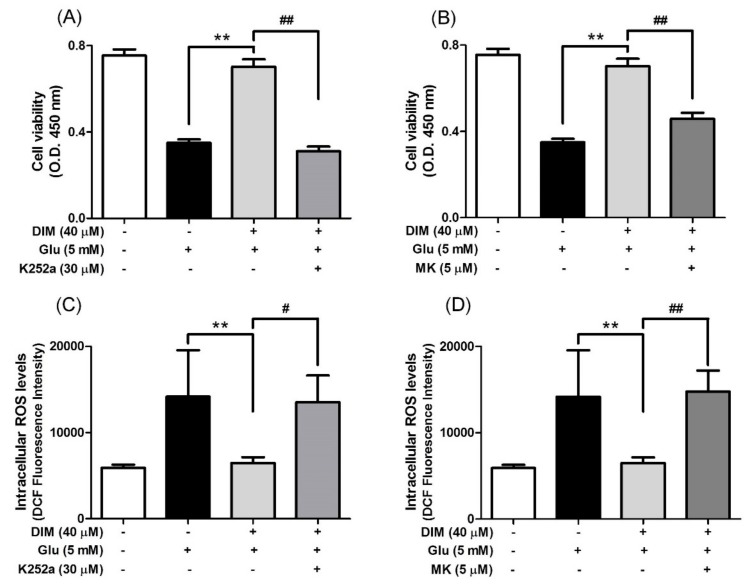
Inhibitory effect of K252a or MK-2206 on neuroprotective action of DIM. HT-22 cells were preincubated with or without DIM in combination with K252a or MK-2206 for 30 min before glutamate challenge. After 12 h, cell viability and ROS level were measured as described in Materials and Methods. (**A**), (**B**) Cell viability and (**C**), (**D**) ROS level. Data are the mean ± SD values of quintuple determinations. ** *p* < 0.01 versus glutamate-treated group; ^#^ *p* < 0.05 and ^##^ *p* < 0.01 versus DIM with glutamate-treated group.

Subsequently, we attempted to clarify the mechanism by which DIM promoted the activation of the TrkB/CREB/BDNF pathway and the Akt/Nrf2/ARE pathway in oxidative stress-exposed hippocampal neuronal cells. For this, we investigated the suppressive effects of K252a, a TrkB inhibitor, and MK-2206, a selective Akt inhibitor, on the neuroprotective action of DIM in oxidative stress-exposed hippocampal neuronal cells. When DIM was preincubated with HT-22 cells in combination with K252a or MK-2206 prior to glutamate treatment, the inclusion of either K252a or MK-2206 significantly attenuated the preventive action of DIM against oxidative stress-induced cell death (Figure 4A,B). In addition, the inhibitors reversed the inhibitory effect of DIM on the generation of ROS in glutamate-treated HT-22 cells (Figure 4C,D). Overall, these results support the notion that DIM protects hippocampal neuronal cells against oxidative stress by promoting the expression of both BDNF and antioxidant enzymes via activation of both the TrkB/CREB/BDNF pathway and the Akt/Nrf2/ARE pathway.

### 3.5. Improving Effect of 3,3′-Diindolylmethane on Scopolamine-Induced Memory Impairment in Mice

**Figure 5:**
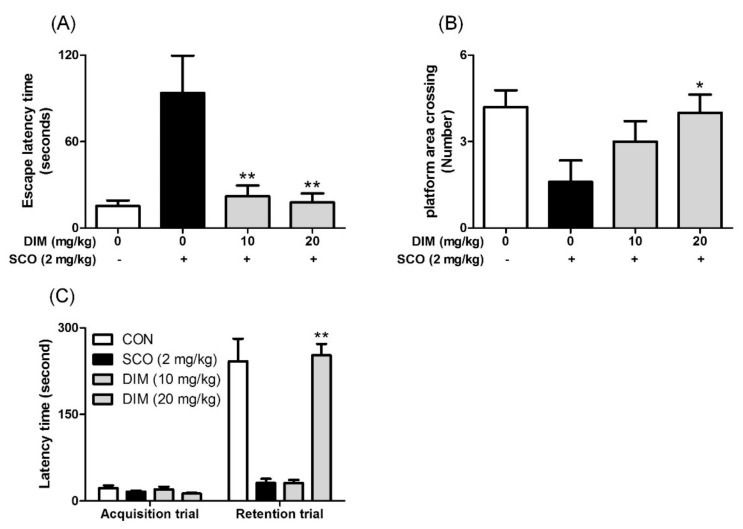
Inhibitory effect of 3,3′-diindolylmethane on memory impairment induced by scopolamine in mice. Mice were orally administrated with DIM (10 or 20 mg/kg) before scopolamine treatment (2 mg/kg, i.p.). After 1 h, the mice were tested for Morris water maze or passive avoidance (Acquisition trial). (**A**) Escape latency time, (**B**) number of platform area crossings, and (**C**) latency time. Data are the mean ± SEM values of sextuple determinations. *P< 0.05 and **P< 0.01 versus scopolamine-treated group.

**Figure 6:**
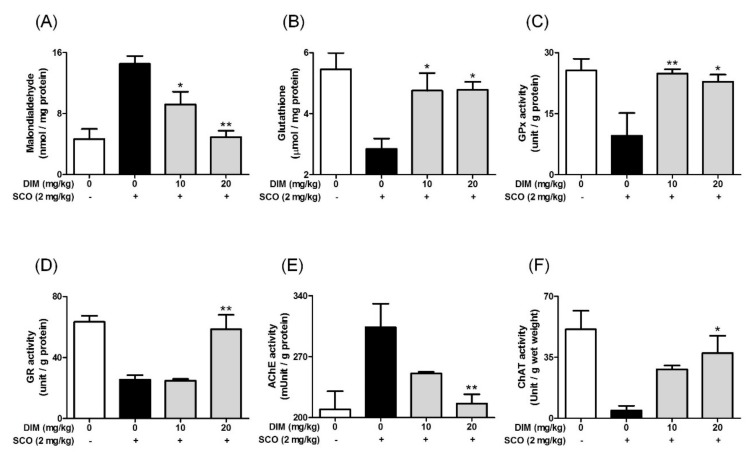
Effect of 3,3′-diindolylmethane on antioxidant biomarkers and cholinergic enzymes in brain tissue of mice treated with scopolamine. Experiments were performed as described in the Figure 5 legend. Lipid peroxidation, GSH, and the activities of GPx, GR, AChE, or ChAT in brain tissues were determined as described in Materials and Methods. (**A**) Lipid peroxidation, (**B**) GSH, (**C**) GPx activity, (**D**) GR activity, (**E**) AChE activity, and (**F**) ChAT activity. Data are the mean ± SEM values of sextuple determinations. * *p* < 0.05 and ** *p* < 0.01 versus scopolamine-treated group.

**Figure 7:**
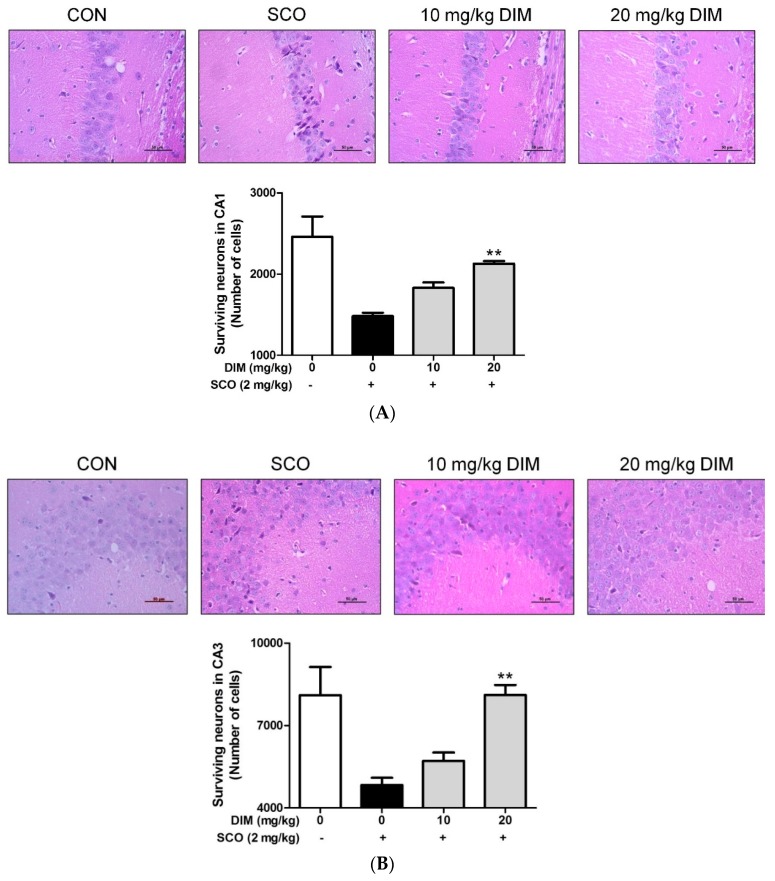
Neuroprotective effect of 3,3′-diindolylmethane on hippocampal CA1 and CA3 regions of mice treated with scopolamine. Experiments were performed as described in the Figure 5 legend. Neuronal cell staining of brain tissues was carried out as described in Materials and Methods. The results were obtained from three independent experiments. (**A**) CA1 region and (**B**) CA3 region. ** *p* < 0.01 versus scopolamine-treated group.

Finally, to confirm the neuroprotective action of DIM in an in vivo system, we further investigated the neuroprotective action of DIM in scopolamine-treated mice. When mice were orally administrated with DIM (10 or 20 mg/kg) before scopolamine challenge, DIM at 20 mg/kg decreased the time of escape latency and increased the number of platform area crossings and latency time in scopolamine-treated mice (Figure 5). However, DIM at 10 mg/kg did not improve the latency time in the mice, which suggests that the administration of DIM at 10 mg/kg failed to reach the effective concentration in brain because of the rapid metabolism of DIM [25]. Consistent with its antioxidant action in an in vitro system, DIM reduced lipid peroxidation and also improved the level of GSH and the activities of GR and GPx in brain tissue (Figure 6A–D). Additionally, DIM reduced AChE activity and enhanced ChAT activity compared with the scopolamine group (Figure 6E,F), further supporting the antioxidant action of DIM. Also, DIM prevented scopolamine-induced oxidative damage of neuronal cells in the hippocampal CA1 and CA3 regions of mice brains (Figure 7). These findings suggest that DIM improves scopolamine-induced memory impairment by protecting neuronal cells against oxidative damage in the hippocampus of mice. Based on this, we suggest that the neuroprotective action of DIM may be largely ascribed to the activation of both the TrkB/CREB/BDNF pathway and the Akt/Nrf2/ARE pathway.

## 4. Discussion

DIM, a dimer of indole-3-carbinol, is known to have some beneficial effects, such as antioxidant action [4], anti-cancer activity [5], hepatoprotection [6], and neuroprotection [7,8]. Recently, it was reported that DIM at 10 μM completely protected neuronal cells against hypoxia through reducing the expression of the aryl hydrocarbon receptor modulator and aryl hydrocarbon receptor induced by hypoxia [7]. In addition, the activation of the aryl hydrocarbon receptor by α-naphthoflavone promoted apoptosis through upregulating ROS generation in neuronal cells [26]. However, DIM could not protect neuronal cells against hypoxia in neuronal cells treated with siRNA targeted to the aryl hydrocarbon receptor modulator or hydrocarbon receptor [7]. Therefore, it remains unknown how DIM protects neuronal cells against oxidative stress-induced apoptosis.

Recently, we reported that *N*-acetyl serotonin protected neuronal cells against oxidative stress-induced apoptosis through activating both the TrkB/CREB/BDNF and Akt/Nrf2/antioxidant enzyme pathways [12], and *N*-palmitoyl serotonin protected neuronal cells against oxidative stress-induced apoptosis by stabilizing the activation of the BDNF autocrine loop, although it could not directly activate the phosphorylation of TrkB [11]. Based on these findings, we surmised that DIM may protect hippocampal neuronal cells against oxidative stress-induced apoptosis by maintaining the activation of both the TrkB/CREB/BDNF pathway and the Akt/Nrf2/ARE pathway. Therefore, we investigated the effect of DIM on oxidative stress-induced apoptosis in hippocampal neuronal cells using an in vitro study. From the results, we found that DIM protected hippocampal neuronal cells against oxidative stress-induced apoptosis by promoting the expression of both BDNF and antioxidant enzymes, such as HO-1, NQO-1, and GCLC. Moreover, in an in vivo study, DIM attenuated scopolamine-induced memory impairment by protecting hippocampal neuronal cells against oxidative damage in mice. Furthermore, such a neuroprotective effect of DIM was possibly associated with the stabilized activation of an antioxidant enzyme-generating system such as the Akt/Nrf2/ARE pathway in hippocampal neuronal cells. These results lead to the assumption that TrkB activation stabilized by DIM may be related to the activation of the Akt/Nrf2/ARE pathway in neuronal cells.

One possible mechanism for the neuroprotective action of DIM may be related to the upregulation of BDNF expression through activation of the TrkB/Akt/CREB/BDNF pathway [18]. BDNF, a ligand of TrkB with a stronger neuroprotective action than that of other neurotrophic factors, is mainly produced from astrocytes [13], microglia [14], and neuronal cells [15]. Here, the stimulation of TrkB by BDNF is known to promote proliferation and differentiation of neural stem cells [17]. In the signaling pathway, the activation of TrkB by BDNF induces the phosphorylation of Akt [27], and then the activated Akt is able to produce BDNF through the activation of CREB in neuronal cells [28]. Thus, maintaining the production of BDNF in neuronal cells may be an important strategy for the prevention or treatment of neurodegenerative diseases, such as Alzheimer’s disease. Consistent with this, our present data indicates that DIM protects hippocampal neuronal cells against oxidative stress-induced apoptosis by elevating the phosphorylation of TrkB, Akt, and CREB. In support of this, the inclusion of K252a, an inhibitor of TrkB, neutralized the neuroprotective effect of DIM on oxidative stress-induced cell death of hippocampal neuronal cells. A similar result was observed previously when HT-22 cells were incubated with glutamate in the presence of *N*-acetyl serotonin [12]. Therefore, we suggest that DIM is able to protect neuronal cells by promoting the formation of BDNF through the activation of the TrkB/Akt/CREB/BDNF pathway in oxidative stress-exposed neuronal cells.

Another possible mechanism for the neuroprotective action of DIM is associated with the formation of antioxidant enzymes such as HO-1, NQO-1, and GCLC through the activation of the Akt/Nrf2/ARE pathway. In support of this, DIM augmented the expression of antioxidant enzymes in accordance with the Nrf2/ARE pathway [29]. Moreover, the action of DIM may be extended to activation of the TrkB/Akt/Nrf2/ARE pathway; DIM activates TrkB to produce p-TrkB as mentioned earlier. In turn, activated Akt liberates Nrf2 from the keap1-Nrf2 complex [12], and then Nrf2 is combined into the ARE region on DNA after it translocates into the nucleus [29]. As a result, Nrf2 promotes the formation of antioxidant enzymes, such as HO-1, NQO-1, and GCLC [29]. In support of this, our present study showed that DIM upregulated the expression of p-TrkB and p-Akt and also improved nuclear translocation of Nrf2. Consequently, DIM restored the level of antioxidant enzymes, such as HO-1, NQO-1, and GCLC, to the control level in oxidative stress-exposed hippocampal neuronal cells. In further support of the TrkB/Akt/Nrf2/ARE pathway, the inclusion of K252a, a TrkB inhibitor, or MK-2206, a selective Akt inhibitor, nullified the suppressive effect of DIM on both ROS generation and cell death in oxidative stress-exposed neuronal cells. Probably consistent with the in vitro results, DIM at low doses (10–20 mg/kg) improved scopolamine-induced memory impairment by protecting neuronal cells against oxidative damage in the hippocampal CA1 and CA3 regions of the brain in mice. Additionally, the cholinolytic effect of scopolamine, mainly due to an increase of AChE activity and a decrease of ChAT activity, was remarkably abrogated by DIM. A similar result was observed previously with *N*-palmitoyl serotonin [19]. A reason for these results is that DIM is capable of crossing to the gastrointestinal tract as well as the blood–brain barrier [25]. In addition, the concentrations of DIM are equal to 0.8 to 1.6 mg/kg (48 to 96 mg/60 kg) in humans, based on body surface area [30].

Taking these results together, it is proposed that the activation of TrkB stabilized by DIM, followed by Akt activation, promotes the expression of both BDNF and antioxidant enzymes in neuronal cells. Therefore, the activation of the TrkB/Akt/Nrf2/ARE pathway, accompanied by stabilization of the TrkB/CREB/BDNF autocrine loop, may be important for the prevention and treatment of neurodegenerative diseases. In addition, the present study shows that DIM may be a candidate compound for the prevention and treatment of neurodegenerative diseases such as Alzheimer’s disease.

## 5. Conclusions

The present study demonstrates that DIM exerts a neuroprotective action by producing BDNF and antioxidant enzymes through activation of the TrkB/Akt signal pathway in oxidative stress-exposed hippocampal neuronal cells. In addition, DIM ameliorates cognitive ability by maintaining the cholinergic system in scopolamine-exposed mice. These findings reveal a feature of the mechanism for the neuroprotective action of DIM against oxidative stress-induced apoptosis in neuronal cells. Such effects of DIM may provide further information for the application of DIM as a neuroprotective agent for the prevention and treatment of neurodegenerative diseases. In addition, this study may be helpful to establish new diagnostic and theranostic paradigms of neurodegenerative diseases, to explore a new biomarker of the diagnostic criteria of neurodegenerative diseases, and to develop innovative pharmacological protocols for the patients of neurodegenerative diseases. Further animal model studies may be required to confirm the feasibility of the use of DIM as a neuroprotective agent for specific neurodegenerative diseases.

## Abbreviations

AChE, acetylcholinesterase; AIF, apoptosis-inducing factor; Akt, protein kinase B; Bax, Bcl-2-associated X protein; Bcl-2, B-cell lymphoma 2; BDNF, brain-derived neurotrophic factor; ChAT, choline acetyltransferase; CREB, cAMP response element-binding protein; DIM, 3,3′-diindolylmethane; GCLC, glutamate-cysteine ligase catalytic subunit; GPx, glutathione peroxidase; GR, glutathione reductase; GSH, glutathione; HO-1, heme oxygenase-1; NQO-1, NAD(P)H quinine oxidoreductase-1; Nrf2, NF-E2-related factor-2; ROS, reactive oxygen species; TrkB, Tropomyosin-related kinase receptor B.
